# Correlation between Bacterial Cell Density and Abundance of Antibiotic Resistance on Milking Machine Surfaces Assessed by Cultivation and Direct qPCR Methods

**DOI:** 10.1007/s00248-023-02225-7

**Published:** 2023-05-11

**Authors:** Mareike Weber, Bettina Göpfert, Sina von Wezyk, Michael Savin-Hoffmeyer, André Lipski

**Affiliations:** 1 Institute of Nutrition and Food Sciences, Department of Food Microbiology and Hygiene, University of Bonn, Friedrich-Hirzebruch-Allee 7, 53115 Bonn, Germany; 2 Institute for Hygiene and Public Health, University Hospital Bonn, Venusberg-Campus 1, 53127 Bonn, Germany

**Keywords:** Dairy microbiota, Surface-associated microbial consortia, Biofilm, Horizontal gene transfer, Antibiotic resistance, Blanket dry cow therapy

## Abstract

The relative abundance of antibiotic-resistant bacteria and antibiotic-resistance genes was surveyed for different parts of a milking machine. A cultivation approach based on swab samples showed a highly diverse microbiota, harboring resistances against cloxacillin, ampicillin, penicillin, and tetracycline. This approach demonstrated a substantial cloxacillin resistance of numerous taxa within milking machine microbiota coming along with regular use of cloxacillin for dry-off therapy of dairy cows. For the less abundant tetracycline-resistant bacteria we found a positive correlation between microbial cell density and relative abundance of tetracycline-resistant microorganisms (R^2^ = 0.73). This indicated an accelerated dispersion of resistant cells for sampling locations with high cell density. However, the direct quantification of the *tetM* gene from the swap samples by qPCR showed the reverse relation to bacterial density if normalized against the abundance of 16S rRNA genes (R^2^ = 0.88). The abundance of 16S rRNA genes was analyzed by qPCR combined with a propidium monoazide treatment, which eliminates 16S rRNA gene signals in negative controls.

**Supplementary Information:**

The online version contains supplementary material available at 10.1007/s00248-023-02225-7.

## Introduction

Antibiotics are not only used in human and veterinary medicine to treat bacterial diseases but also by dairy farms for routine dry-off of dairy cows. This treatment prevents intramammary infections following the lactation period [1]. Resistance mechanisms against antibiotics are manifold and often located on horizontally transferable mobile genetic elements (MGE). Mechanisms may be rather unspecific, such as multi-drug efflux pumps, or more specific, such as ribosomal protection proteins (e.g. coded by *tetM*), or β-lactamases that cleave β-lactam antibiotics (e.g. coded by *blaZ* or OXA) [2, 3].

The selective pressure resulting from extensive antibiotic use in dairy production can trigger the proliferation of (multi-) resistant microorganisms [4]. Environmental microorganisms harbor a wide variety of antibiotic resistance genes. These organisms might act as a reservoir of resistance genes, which can be transferred to human or animal pathogens, such as mastitis pathogens, under appropriate conditions [5, 6]. The presence of surface-associated microbial consortia within milking machines is a well-known phenomenon [7] and it is suspected that dense microbial communities show enhanced horizontal gene transfer (HGT) [8]. The spatial proximity of surface-associated cells facilitates conjugation, and external DNA (eDNA) as a structural component of the extracellular polymeric substances (EPS) may trigger competence development for transformation [9]. Dense bacterial communities of high diversity are supposed to be appropriate habitats for the distribution of antibiotic resistance genes across species boundaries.

Antibiotic resistant consortia can be assessed by cultivation or direct detection of resistance genes by qPCR to avoid cultivation steps. Cultivation methods rely on distinct antibiotic concentrations in culture media to differentiate between sensitive and resistant bacteria [10, 11]. The thresholds listed in European (EUCAST) [10] and international reference tables (CLSI M100 [11] and Vet01) refer to pure cultures of pathogens relevant for human and veterinary medicine, and antibiotic classes that are relevant for the treatment of the respective disease. Thresholds for environmental organisms are not included.

For the bacterial communities of the milking machine analyzed in this project we assumed that the frequent use of antibiotics on the dairy farm exerts selective pressure triggering the proliferation of antibiotic resistant bacteria. Moreover, we assumed that the dispersion of antimicrobial resistance genes is more efficient in dense bacterial communities. To test this assumption, we related the abundance of resistant cells and resistance genes to the cell density at the sampling sites. The proportion of antibiotic resistant bacteria was expected to be higher in densely populated microbial consortia because mechanisms such as horizontal gene transfer may be intensified in microbial communities with higher cell concentrations.

## Materials and Methods

### Characterization of the Investigated Dairy Farm

The samples were taken from a dairy research farm of the University of Bonn in Königswinter, Germany (L: 7° 12′ 22″ E, B: 50° 42′ 49″ N). A detailed description of the dairy farm as well as a schematic diagram of the milking machine can be found in Weber et al*.* (2019) [7].

### Cultivation Methods

Swab samples were taken from different parts of the milking machine. Three samplings took place in total (further referred to as samplings A, B, C) in June and December 2015, and May 2016. The sampling locations were: the milking equipment retainers (R), the separator (S), the filter pipe at the beginning of the pressure line (FP), the plastic pipe at the beginning of the pressure line (BP), the stainless steel pipe at the end of the pressure line (ESS), the plastic pipe at the end of the pressure line (EPP), the outlet of the raw milk bulk tank (OB), and the bulk tank interior (BI). Swabs were transferred into 10 ml of Ringer’s solution, and stirred on a vortex mixer for 25 s. Decimal dilutions were plated on the following media: tryptic soy agar (TSA) (Merck, Darmstadt, Germany) for total viable count (TVC), as well as TSA containing different antibiotics for resistant microbial counts (concentrations see Table 1): cloxacillin sodium salt (clox) (Alfa Aesar/Thermo Fisher Scientific, Kandel, Germany), ampicillin sodium salt (amp), penicillin G sodium salt (pen) (both from Carl Roth, Karlsruhe, Germany), and tetracycline hydrochloride (tet) (Sigma Aldrich/Merck, Darmstadt, Germany). In sampling A, de Man, Rogosa, and Sharpe (MRS) agar (Merck, Darmstadt, Germany) and MRS agar containing the antibiotics listed above under anaerobic incubation was additionally used to detect lactic acid bacteria (LAB).

**Table 1:** Antibiotic concentrations in tryptic soy agar (TSA) used in the three samplings A, B, and C

Sampling	Concentration (μg/ml) in TSA			
	Cloxacillin	Ampicillin	Penicillin	Tetracycline
A	1	8	1	4
B	2	16	16	8
C	4	16	16	8

### Differentiation and Identification of Isolates

The dominating colony morphologies from antibiotic-containing TSA were selected for isolation. Isolates were grouped according to their fatty acid profiles, and representatives of each group were identified by 16S rRNA gene sequencing as described previously [7]. Selected isolates were cultivated on TSA media containing all four antibiotics to determine cross-resistances. Isolates were combined as operational taxonomic units (OTU) at ≥ 99% sequence identity. A phylogenetic tree was constructed using the program MEGA 11 [12] with representative sequences from each OTU or single species. We used the Maximum Likelihood algorithm with the Kimura-2 parameter model, Gamma distributed rates with invariant sites (G + I), and a partial deletion mode with a cut-off value of 95%. A bootstrap test with 1,000 replications was used to test the phylogeny. The 16S rRNA gene sequences created in this study are deposited in Genbank under the accession numbers OP018821-OP018861.

### Swab Sample DNA Extraction

Cell pellets were produced from 2 × 2 ml of the swab samples suspended in Ringer’s solution by centrifugation (Eppendorf Centrifuge 5417 R, Hamburg, Germany) for 10 min at full speed (13,000 × g). DNA was extracted using the DNeasy Blood & Tissue Kit (Qiagen, Hilden, Germany) according to the manufacturer’s recommendations for Gram-negative and Gram-positive bacteria. Gram-positive bacteria were extracted by resuspending the cell pellet in 180 µl of lysis buffer [20 mM Tris HCl (pH 8.2), 2 mM EDTA, and 1.2% Triton X-100] containing 20 mg/ml lysozyme as described earlier [7]. For Gram-negative bacteria, the cell pellet was resuspended in 180 µl buffer ATL and 25 µl Proteinase K, and incubated at 55 °C overnight. Samples were vortexed, 200 µl buffer AL was added, and incubated at 70 °C for 30 min. 200 µl of ethanol was added to all samples, and DNA was extracted according to the manufacturer’s instructions. DNA was eluted in two steps by adding 100 µl ddH_2_O to the columns. The extract was concentrated to half of the initial volume using a vacufuge (5301, Eppendorf, Hamburg, Germany).

### Realtime Quantitative PCR (qPCR)

16S rRNA- and antibiotic resistance genes OXA, *blaZ*, and *tetM* were amplified from 2 µl swab DNA extracts and DNA extracts of reference strains using the QuantiTect SYBR Green PCR Mastermix Kit (Qiagen, Hilden, Germany). Primer pairs are listed in Supplementary Table 1. Amplificate length was verified using 2% agarose gels, selected PCR products were sequenced, and melting curves were generated in every qPCR-reaction. Using the mastermix with universal 16S rRNA gene primers, signals were detected also in no template controls (NTCs). To suppress unspecific signals, we combined the method described by Hein et al*.* (2007) [13] with our previously described method [14] using propidium monoazide (PMA) for treating the mastermixes used for 16S rRNA gene amplification prior to template addition. Mastermixes were prepared and aliquoted to the final volume. To each aliquot 1 µl PMA [20 mM stock solution in 20% DMSO, 12.5 to 25 µM working solutions in ddH_2_O] was added resulting in final concentrations ranging from 0.625 to 1.25 µM PMA. The aliquots were incubated for 20 min at 4 °C in the dark, and then activated by light exposure with a 400 W halogen lamp at a distance of 25 cm. To avoid overheating, the tubes were kept on ice during light exposure. We used a final concentration of 0.8 µM PMA in the mastermixes for 16S rRNA gene amplification of swab samples. Mastermixes for antibiotic resistance gene amplification were used without PMA treatment. The PCR program for each qPCR reaction consisted of a first denaturation step at 95 °C for 15 min, followed by 35 cycles (16S rRNA genes), or 40–45 cycles (antibiotic resistance genes) of denaturation at 95 °C for 15 s, primer annealing for 30 s at 57 °C (16S rRNA), 52,1 °C (OXA-genes), 50 °C (*blaZ*), and 47 °C (*tetM*), respectively, and elongation at 72 °C for 30 s.

### Quantification of 16S rRNA- and Antibiotic Resistance Genes

For relative quantification, the cycle threshold (C_T_) for antibiotic resistance genes was divided by the C_T_ for 16S rRNA genes. For absolute quantification of 16S rRNA genes, we amplified the nearly full-length 16S rRNA genes (1500 bp) of the reference strains *E. coli* E2 and *S.* *epidermidis* RP26A using primer pair GM3F (5 ‘-AGAGTTTGATCMTGGC-3 ‘) and GM4R (5 ‘-TACCTTGTTACGACTT-3 ‘) [15]. Assuming that the purified PCR products contain only 16S rRNA amplicons of 1500 bp size, we used Formula 1 after Whelan et al*.* (2003) [16] to calculate 16S rRNA gene copies/µl PCR product:

1 $$\frac{\mathrm{g}\mathrm{ene}\ \mathrm{copies}}{\upmu \mathrm{l}}=\frac{\mathrm{DNA}\ \mathrm{conc}.\kern0.5em \left(\ \frac{\mathrm{g}}{\upmu \mathrm{l}}\right)\bullet 6.02\bullet {10}^{23}\ \left(\ \frac{\mathrm{mol}\bullet \mathrm{Da}}{\mathrm{g}}\right)}{\mathrm{amplicon}\ \mathrm{size}\ \left(\mathrm{bp}\right)\bullet 660\ \left(\frac{\mathrm{Da}}{\mathrm{bp}\bullet \mathrm{mol}}\right)}$$

Decimal dilutions of the purified PCR products were used to amplify an inner fragment of the 16S rRNA gene via qPCR using the primer pair F1048/R1194 (Suppl. Tab. 1). A standard curve was created using linear regression. The concentration of 16S rRNA genes in samples with unknown composition was calculated via their C_T_ value. For absolute quantification of antibiotic resistance genes, purified PCR products created with the primer pairs listed in Suppl. Tab. 1 were cloned into the pGEM®-T Vector (Promega, Madison, USA) according to the manufacturer’s instructions. Competent *E. coli* JM109 cells (Promega, Madison, USA) were transformed with the vector, DNA of vector-containing clones was extracted and used as a template for PCR amplification with the primer pair M13F (5 ‘-GTTTTCCCAGTCACGAC-3 ‘) and M13R (5 ‘-CAGGAAACAGCTATGAC-3 ‘) [17] as described earlier [7]. The primer pair amplifies the respective insert plus about 200 bp of the vector. Standard curves were created by qPCR as described for 16S rRNA genes. For comparison with data from the cultivation approach, we calculated total bacterial count (TBC) equivalents using the amount of 16S rRNA genes calculated from the standard curves. For this calculation we used the average number of 4.2 16S rRNA gene copies per bacterial cell as proposed by Větrovský & Baldrian (2013) [18] to provide TBC equivalents per cm^2^ sampled area. Proportions of antibiotic resistance genes were calculated per TBC equivalent.

## Results

### Composition of Antibiotic Resistant Milking Machine Microbiota

**Fig. 1:**
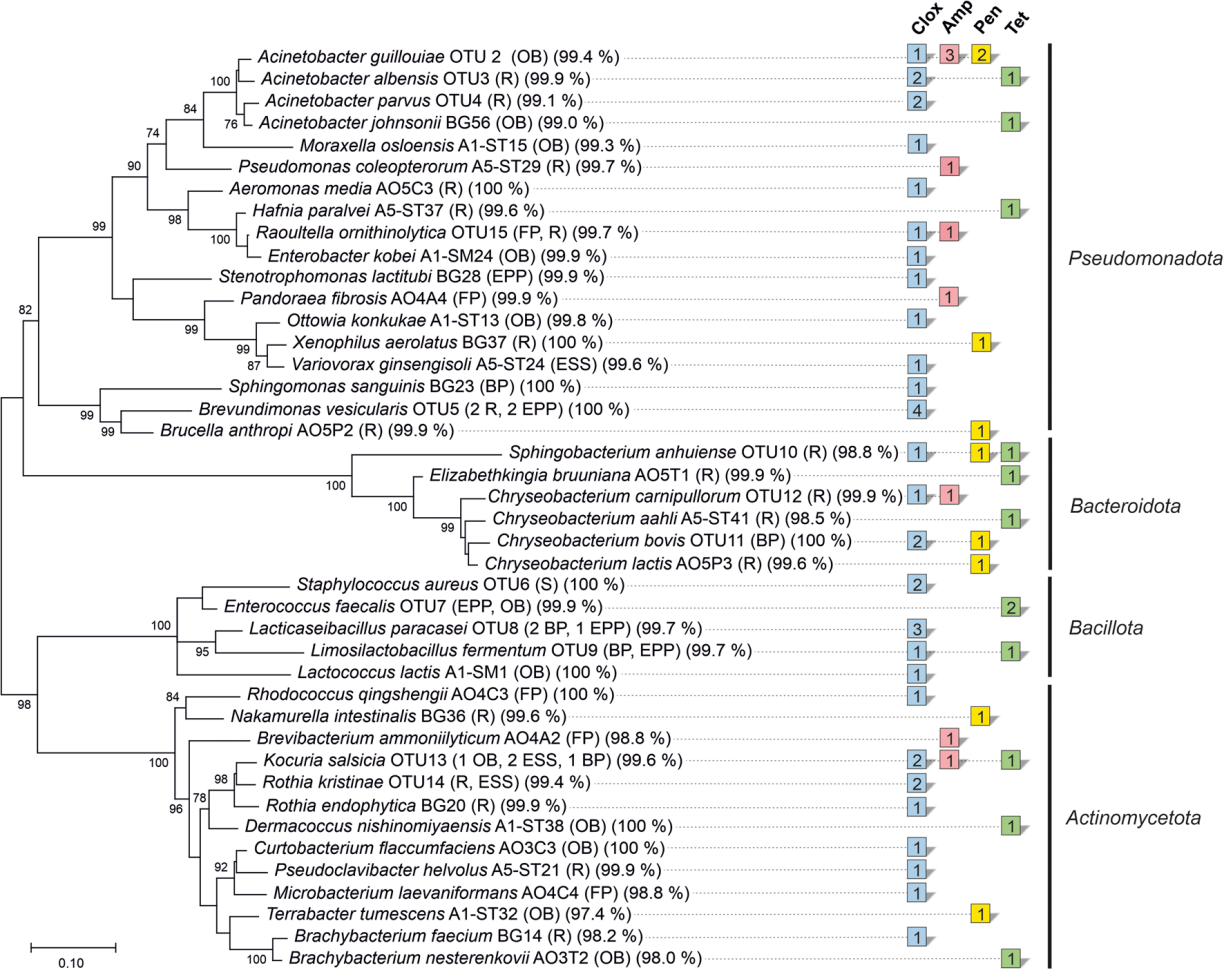
Maximum Likelihood phylogenetic tree of antibiotic resistant isolates from milking machine surfaces. The sampling site as well as the 16S rRNA gene sequence percent identity with the type strain of the respective species is given in parentheses. Isolates were assigned to the same operational taxonomic unit (OTU) at ≥ 99% sequence identity. Different antibiotics are represented by colored squares in different columns. The number of isolates is indicated inside each square. Bootstrap values ≥ 70% from 1,000 replicates are indicated on the branches

A high diversity of antibiotic resistant bacteria from the phyla *Actinomycetota*, *Bacteroidota*, *Bacillota*, and *Pseudomonadota* was detected (Fig. 1). Dominant phyla with high species diversities were *Pseudomonadota* with 30 isolates from 15 genera, and *Actinomycetota* with 20 isolates from 11 genera. The phylum *Bacteroidota* was represented by 11 isolates from 3 genera, while the phylum *Bacillota* was represented by 10 isolates of 5 species and genera, respectively. Due to high resistant bacterial counts on cloxacillin-TSA, the majority of 40 isolates originated from cloxacillin-TSA, while 9 isolates originated from ampicillin- and penicillin-TSA, respectively. 13 strains were isolated from tetracycline-TSA. The genera *Acinetobacter* and *Chryseobacterium* displayed the highest species diversities with four different species each, represented by nine and seven isolates, respectively. The isolates originated from TSA containing all four of the tested antibiotics, pointing to a high level of resistance in these genera. The phylum *Bacillota* was mainly represented by lactic acid bacteria (LAB). Seven out of eight lactic acid bacteria isolates originated from antibiotic-containing MRS agar.

The majority of isolates showed resistance against all four antibiotics used in this study at their highest concentration (Supplementary Table 2). Many isolates exhibited resistance against one or more β-lactam antibiotic, and additionally against tetracycline. The majority of isolates was resistant to cloxacillin. Resistance against cloxacillin often, but not always, occurred simultaneously to resistance against ampicillin and penicillin. *Brachybacterium* spp. AO3T2 was the only isolate resistant to tetracycline while sensitive to all three β-lactam antibiotics.

### PMA Treatment to Suppress Unspecific qPCR Signals

**Fig. 2:**
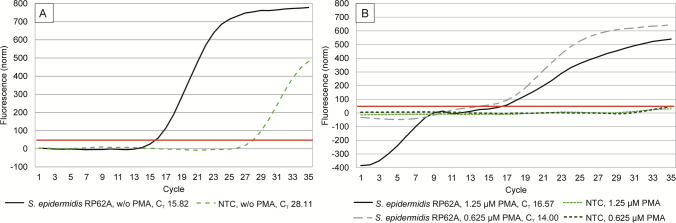
Amplification plots and cycle thresholds (C_T_) of 16S rRNA genes of the positive control *Staphylococcus epidermidis* RP62A and the no template control (NTC) using universal primers without (w/o) treating the mastermix with propidium monoazide (PMA) prior to template addition (**A**), and after treating the mastermix with 0.625 µM and 1.25 µM PMA prior to template addition (**B**)

In the 16S rRNA gene quantification by qPCR the NTC of the untreated mastermix produced a C_T_ of 28, while the positive control *Staphylococcus epidermidis* RP62A produced a C_T_ of 16 (Fig. 2A). This background signal of 16S rRNA qPCR analyses was effectively reduced by PMA treatment of the mastermixes. Both PMA concentrations effectively suppressed NTC signals for 35 cycles (Fig. 2B). However, a PMA concentration of 1.25 µM produced an irregular shape of the amplification plot of the positive control, while a lower PMA concentration of 0.625 µM had a flatter curve progression compared to the untreated mastermix, but the C_T_-value was nearly unaffected by PMA treatment. A qPCR mastermix supplementation with 0.625 µM PMA improved the detection limit for TBC equivalents calculated from 16S rRNA gene amplification by more than 1.7 log-levels.

### Comparison of Antibiotic Resistant Counts Determined by Cultivation and qPCR

The abundance of antibiotic resistant counts based on cultivation and on qPCR analysis are presented in Table 2. No qPCR signals were detected for *blaZ*, OXA-1, and OXA-10 genes. Signals for *tetM* genes were detected for almost every sample. In contrast to the high percentages of cloxacillin resistant microorganisms detected by cultivation, OXA-2 genes were only detected on the milking equipment retainers of samplings A and C (Tab. 2). The milking equipment retainers displayed the highest population density of around 7.5 log_10_ cfu/cm^2^ in all three samplings (Tab. 2). In contrast, population densities of the pipe system and the outlet of the milk bulk tank varied significantly between different samplings. Cultivation counts on MRS agar determined in sampling A were 1–2 log-levels lower than the corresponding counts on TSA, while TBC equivalents determined by qPCR were 1–2 log-levels higher than the corresponding TVCs on the same sampling spot in most cases. The TBC-equivalents determined for the pipe system of the milking machine were remarkably higher than the respective cultivation counts. For example, TVCs were lower than the detection limit of 0.5 log cfu/cm^2^ for samplings B and C, while the corresponding TBC-equivalents determined by qPCR were 4.9, and 3.3 cfu-equ./cm^2^, respectively.

**Table 2:**
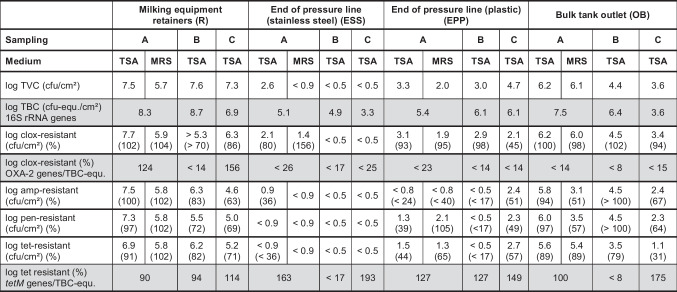
Total viable counts (TVC) and antibiotic resistant microbial counts on TSA and MRS agar containing cloxacillin (clox), ampicillin (amp), penicillin (pen), and tetracycline (tet) for the four sampling spots investigated in all three samplings A, B, and C. Percentage of log resistant microbial counts are given in parentheses for each sampling

Total bacterial count (TBC) equivalents determined by qPCR amplification of 16S rRNA genes as well as proportion of OXA-2- and *tetM*-genes in reference to TBC-equivalents on the same sampling areas are highlighted by grey shadings

For most surfaces, TVC and cloxacillin resistant counts were in the same range. The proportions of ampicillin, penicillin and tetracycline resistant microorganisms often exceeded 60% of TVC. The plastic pipe at the end of the pressure line (EPP) was the surface harboring the lowest proportion of antibiotic resistant microorganisms. Despite of constant antibiotic concentrations used for cultivation of ampicillin, penicillin and tetracycline resistant microorganisms in samplings B and C, varying proportions of resistant microbial counts were detected. For example, the proportion of tetracycline resistant microorganisms on the bulk tank outlet (OB) was 80% in sampling B, while it was only 31% in sampling C. In most cases, antibiotic resistant percentages on MRS agar matched with the corresponding percentages on TSA. Higher proportions of resistant microorganisms on MRS agar compared to TSA were detected on the plastic pipe at the end of the pressure line (EPP). While 39% and 44% were rated as penicillin and tetracycline resistant, respectively, on TSA, resistant proportions on MRS agar were 105% and 65%.

The abundance of *tetM*-genes was close to or even higher than the corresponding TBC-equivalents, resulting in percentages larger than 90%. The difference between cultivation and qPCR amplification of *tetM* genes was especially striking for the pipe system of the milking machine. The percentage of tetracycline resistant microorganisms determined by cultivation on TSA reached a maximum of 57% on the plastic pipe (EPP) in sampling C. In contrast, the percentage of *tetM*-genes determined by qPCR was 149%. Although TBC-equivalents detected on the stainless steel pipe in samplings A and B were similar to each other (5.1 and 4.9 log_10_ cfu-equ./cm^2^), the percentage of *tetM*-genes varied greatly from 163% to not detectable (< 17%).

### Relationship between Microbial Cell Density and Abundance of Antibiotic Resistance

**Fig. 3:**
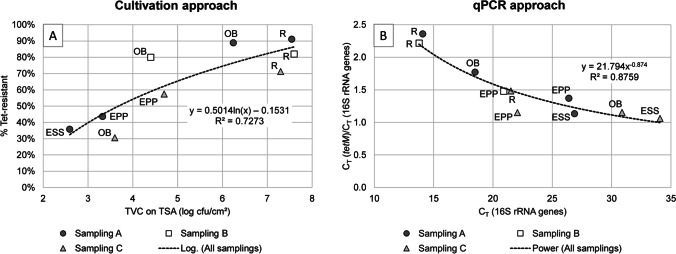
Correlation between microbial cell density in milking machine associated microbial consortia and abundance of tetracycline (tet) resistance as assessed by the cultivation approach (**A**) and the qPCR amplification of 16S rRNA- and *tetM*-genes (**B**). Regression and R^2^ of the curves were calculated by Excel 2016. The curve-fitting model displaying the highest R^2^ was used to describe the correlation between microbial cell density and abundance of antibiotic resistance. A: Total viable counts (TVC) determined on tryptic soy agar (TSA) in relation to percent tetracycline resistant microorganisms determined on tetracycline-containing TSA on different parts of the milking machine. B: Cycle threshold (C_T_)-values of 16S rRNA genes determined by qPCR amplification in relation to the ratio of C_T_-values of *tetM*- and 16S rRNA-genes on different parts of the milking machine. Values from samplings A, B, and C are indicated by different symbols. The sampled part is indicated by abbreviation next to its symbol: R, milking equipment retainers; ESS, stainless steel pipe at the end of the pressure line; EPP, plastic pipe at the end of the pressure line; OB, outlet of the milk bulk tank

A positive correlation between microbial cell density and percentage of tetracycline resistant microorganisms was detected in the cultivation approach. The correlation was described best with a logarithmic regression (R^2^ = 0.73), which indicated an approximation to 100% tetracycline resistant cells with increasing cell density (Fig. 3A). The milking equipment retainers (R) of all three samplings had the highest population densities of about 7.5 log_10_ cfu/cm^2^ and harbored high percentages of tetracycline resistant microorganisms with > 70% resistant cells. In contrast to the cultivation based data the molecular approach depicted in Fig. 3B revealed a negative correlation between microbial cell density and percentage of tetracycline resistant bacteria (R^2^ = 0.88). Based on *tetM* genes detected by qPCR, the percentage of tetracycline resistant bacteria was higher on areas with low abundance of 16S rRNA genes and vice versa.

## Discussion

### Diversity and Antibiotic Resistance Profile of Milking Machine Microbiota

The antibiotic resistant microbiota isolated from milking machine surfaces was highly diverse. This is in accord with previous studies of the same milking machine [7], raw milk of the same dairy farm [14], and other studies investigating the microbiota of raw milk and dairy surfaces [19, 20]. The isolation of different resistant strains of the genera *Acinetobacter* and *Chryseobacterium* points to a key role of these Gram-negative taxa in the antibiotic resistant microbiota of milking machines. The species *Staphylococcus aureus* is a mastitis pathogen, and was isolated twice from TSA containing cloxacillin. Cloxacillin-benzathin (2:1) is the active agent in the preparation used for dry-off (Orbenin® Extra, Zoetis GmbH, Berlin, Germany) on the investigated dairy farm. Cloxacillin resistance of mastitis pathogens is a health risk for the dairy herd. The high level of cloxacillin resistance detected in this study as well as the dissemination of cloxacillin resistance over a wide variety of bacterial taxa indicates the constant selective pressure resulting from its preventive use at dry-off. While it is undoubted that extensive antibiotic use triggers the proliferation of antibiotic resistant bacteria, there is currently no evidence that veterinary use of antibiotics leads to enhanced resistance in veterinary pathogens [4, 21]. If antibiotic dry-off is inevitable, the antibiotic agent should be exchanged in regular intervals to minimize selective pressure. Following the One Health concept the antibiotic agent applied for dry-off should not belong to the group of critically important antibiotics in human medicine listed by the World Health Organization [6]. Many isolates displayed resistance against more than one of the four antibiotics tested in this study. Penicillin G, ampicillin and tetracycline are used less frequently than cloxacillin on the investigated dairy farm, and only for therapeutic purposes. Co-selection of resistance genes against β-lactam antibiotics and tetracycline may have taken place in the current study [22].

### Proportion of Antibiotic Resistant Microorganisms Assessed by Cultivation

Resistance against ampicillin and penicillin was less pronounced than cloxacillin resistance. This is unexpected since many OXA-β-lactamases conferring resistance to cloxacillin should also be able to cleave ampicillin and penicillin [23]. One possible reason for the high percentage of cloxacillin resistant microorganisms could be inappropriate (i.e. too low) cloxacillin concentrations used for the selective detection of resistant microbiota by cultivation. The recommended MIC for oxacillin is 0.25 µg/ml for *Staphylococcus spp.* [10], which is a quarter of the lowest cloxacillin concentration used in this study. Either CLSI M100 [11] nor EUCAST [10] list separate MICs for cloxacillin. Another explanation for the discrepancy between resistance against cloxacillin and ampicillin/penicillin is the presence of highly specific resistance mechanisms against different groups of β-lactam antibiotics. Resistance against cloxacillin combined with sensitivity against ampicillin and penicillin was observed for many isolates (Suppl. Tab. 2), indicating specific resistance mechanisms. In spite of the different modes of action compared to β-lactam antibiotics, tetracycline also produced high resistant counts. Tetracycline is hardly biodegradable and has a long half-life, making it conceivable that this antibiotic exerts selective pressure on bacteria for long periods after its release into the environment [24, 25]. An advantage of cultivation of the entirety of bacteria being able to produce colonies on antibiotic-containing TSA is that no further knowledge of the underlying resistance mechanisms is necessary, making this method an appropriate starting point for further studies.

### Comparability of Cultivation Counts and TBC Equivalents Determined by qPCR

We modified the qPCR protocol using universal 16S rRNA gene primers due to positive signals in the NTCs, which is a well-known phenomenon [13]. Low detection limits are required for quantifying 16S rRNA genes in swab samples with low population densities resulting in low DNA concentrations [26, 27]. These were the majority of samples, especially the pipe system of the milking machine, with C_T_ –values of up to 32. The ideal PMA concentration that suppresses NTC signals while exerting minimal impact on the sensitivity of the qPCR reaction had to be determined for every lot of mastermix kit.

TBC equivalents calculated from qPCR quantification of 16S rRNA genes were higher than the corresponding TVCs determined by cultivation. This is not surprising, since surface associated microbial consortia harbor viable but nonculturable (VBNC) cells detectable by qPCR but not by cultivation [28]. Moreover, not all bacterial groups are able to grow on TSA. Lactic acid bacteria (LAB) are preferentially detected by anaerobic cultivation on MRS agar [9]. Despite of the low cultivation counts on tetracycline containing TSA and MRS agar, high amounts of *tetM* genes were detected in pipe system of the milking machine by qPCR. The species *Enterococcus faecalis*, in addition to other LAB species, was isolated twice, indicating the presence of LAB in the pipe system. Enterococci, especially *Ec. faecalis*, are often associated with tetracycline resistance [29]. *TetM* genes are frequently detected in enterococci, and are often associated with MGE [30–32].

Contrasting the high percentage of cloxacillin resistant microorganisms detected by the cultivation approach, OXA-2 genes were solely detected on the milking equipment retainers in two samplings. This highlights a disadvantage of the qPCR approach. A certain knowledge of which resistance genes are expected to be present within DNA extracts is needed to specifically target them by qPCR. Milking machines harbor a high diversity of mainly environmental bacteria [7], resulting in a variety of possible antibiotic resistance genes. Environmental bacteria are a relevant reservoir of resistance genes transferrable via HGT. For this reason, many authors stress the need to determine the resistome, i.e. the entirety of resistance genes, of habitats of interest [2, 33, 34]. Frequently occurring resistance genes can then be targeted by qPCR.

### Correlation between Bacterial Cell Density and Abundance of Antibiotic Resistance

The working hypothesis of this study was that high cell densities in surface-associated microbial consortia are associated with increased abundance of antibiotic resistant bacteria/antibiotic resistance genes, because horizontal transfer of antibiotic resistance genes is intensified with increased cell density. While the cultivation approach clearly demonstrates the positive correlation between percentage of tetracycline resistant bacterial cells and cell density, which is in accord with our working hypothesis, analyses of *tetM* abundance did not support our results from the cultivation approach. The normalized abundance of *tetM* showed a negative correlation with the abundance of 16S rRNA genes. There are some basic differences between both approaches, which may be causative for this contrastive observation. While the range of 16S rRNA gene copy numbers for bacterial species is known and can be used for calculation [18], the number and range of *tetM* genes per cell is speculative. The gene can be located on the chromosome or be plasmid coded [35], can be part of bacteriophage DNA [36] or even part of the extracellular DNA pool [37]. These variables may mask the correlation expected from the cultivation approach.

In this study we were able to prove the correlation between the use of cloxacillin for dry-off and a high abundance of cloxacillin resistant bacteria in milking machine associated microbial consortia. For tetracycline resistance we found a positive correlation between bacterial cell densities and abundance of antibiotic resistant cells based on a phenotypic, cultivation-based approach. We successfully applied PMA to the mastermix prior to 16S rRNA gene amplification using universal primers. This is an important step towards lowering the detection limit in samples with low bacterial counts. Due to the different advantages of cultivation and qPCR methods discussed above, we recommend applying a cultivation-based approach combined with next-generation metagenome sequencing to determine the resistome of food associated microbial consortia with high, but mostly unknown bacterial diversity. Abundant resistance genes can be quantified by qPCR in a subsequent step.

## Supplementary Information

Below is the link to the electronic supplementary material.

Supplementary file1 (DOCX 23.6 KB)
