# Supplementary material for: Correlation between Bacterial Cell Density and Abundance of Antibiotic Resistance on Milking Machine Surfaces Assessed by Cultivation and Direct qPCR Methods
Source: Microb Ecol. 2023 May 11;86(3):1676–85. doi: 10.1007/s00248-023-02225-7 (PMC10497690; doi:10.1007/s00248-023-02225-7)
Supplement: Supplementary file 1 — Supplementary file1 (DOCX 23.6 KB) [file 248_2023_2225_MOESM1_ESM.docx]

**Supplementary Material**

**Correlation between bacterial cell density and abundance of antibiotic resistance on milking machine surfaces assessed by cultivation and direct qPCR methods**

Mareike Weber, Bettina Göpfert, Sina von Wezyk, Michael Savin-Hoffmeyer, André Lipski ^1)^

^1)^ Corresponding author: lipski@uni-bonn.de

**Supplementary Table 1:** PCR-Primers and reference strains used for qPCR-detection of 16S rRNA- and antibiotic resistance genes.

| **Gene** | **Name** | **Primer sequence (5’ 🡪 3’)** | **Amplicon size (bp)** | **Source** | **Reference strain** |
| --- | --- | --- | --- | --- | --- |
| **16S rRNA** | F1048 | GTGSTGCAYGGYTGTCGTCA | 146 | [1] | *Escherichia coli* E2,  *Staphylococcus epidermidis*  RP62A |
|  | R1194 | ACGTCRTCCMCACCTTCCTC |  |  |  |
| ***blaZ*** | BG-Fneu | GAGATTTGCCTATGCTTCRACT | 233 | [2], modified | *S. epidermidis*  RP62A |
|  | BG-R | CCACCGATYTCKTTTATAATTT |  |  |  |
| ***tetM*** | tetM-FW | ACAGAAAGCTTATTATATAAC | 171 | [3] | *S. aureus* 1450194 |
|  | tetM-RV | TGGCGTGTCTATGATGTTCAC |  |  |  |
| **OXA-1** | B1-F | TTTTCTGTTGTTTGGGTTTT | 427 | [4] | *E. coli* E2 |
|  | B1-R | TTTCTTGGCTTTTATGCTTG |  |  |  |
| **OXA-2** | B2-F | AAGAAACGCTACTCGCCTGC | 478 |  | *E. coli* W3110 |
|  | B2-R | CCACTCAACCCATCCTACCC |  |  |  |
| **OXA-10** | B10-F | TCAACAAATCGCCAGAGAAG | 276 |  | *Providencia stuartii* P125 |
|  | B10R | TCCCACACCAGAAAAACCAG |  |  |  |

[1] Maeda H, Fujimoto C, Haruki Y, Maeda T, Kokeguchi S, Petelin M, Arai H, Tanimoto I, Nishimura F, Takashiba S (2003) Quantitative realtime PCR using TaqMan and SYBR Green for *Actinobacillus actinomycetemcomitans*, *Porphyromonas gingivalis*, *Prevotella intermedia*, *tetQ* gene and total bacteria. FEMS Immunol Med Mic 39:81-86. https://doi.org/10.1016/S0928-8244(03)00224-4

[2] Pereira L, Harnett G, Hodge M, Cattell J, Speers D (2014) Real-time PCR assay for detection of *blaZ* genes in *Staphylococcus aureus* clinical isolates. J Clin Microbiol 52 (4):1259-1261. https://doi.org/10.1128/JCM.03413-13

[3] Aminov R, Garrigues-Jeanjean N, Mackie R (2003) Molecular ecology of tetracycline resistance: Development and validation of primers for detection of tetracycline resistance genes encoding ribosomal protection proteins. Appl Environ Microb 67(1):22-32. https://doi.org/10.1128/AEM.67.1.22-32.2001

[4] Bert F, Branger C, Lambert-Zechovsky N (2002) Identification of PSE and OXA β-lactamase genes in *Pseudomonas aeruginosa* using PCR–restriction fragment length polymorphism. J Antimicrob Chemother 50(1):11-18. <https://doi.org/10.1093/jac/dkf069>

**Supplementary Table 2:** Growth of selected isolates on tryptic soy agar (TSA) containing all four antibiotics (cloxacillin, clox; ampicillin, amp; penicillin, pen; tetracycline, tet) in the lowest and highest concentration used to determine total resistant microbial counts in different samplings of this study. The initial isolation medium is indicated (*). If isolates grew on TSA containing the respective higher antibiotic concentration, growth on the lower concentration of the same antibiotic was not determined (nd). The extent of growth is indicated as follows: ++, strong growth; +, moderate growth; (+) weak growth; − no growth.

| **Isolate** | **TSA**  **+ Clox (µg/ml)** | | **TSA**  **+ Amp (µg/ml)** | | **TSA**  **+ Pen (µg/ml)** | | **TSA**  **+ Tet (µg/ml)** | |
| --- | --- | --- | --- | --- | --- | --- | --- | --- |
|  | **1** | **4** | **8** | **16** | **1** | **16** | **4** | **8** |
| *Microbacterium* spp. AO4C4 | nd | ++* | nd | (+) | (+) | − | nd | ++ |
| *Curtobacterium flaccumfaciens* AO3C3 | nd | +* | (+) | (+) | + | − | − | − |
| *Terrabacter* spp. A1-ST32 | nd | + | + | − | ++* | − | nd | ++ |
| *Brachybacterium* spp. BG14 | nd | +* | (+) | − | + | − | nd | ++ |
| *Brachybacterium* spp. AO3T2 | (+) | − | − | − | − | − | nd | ++* |
| *Brevibacterium* spp. AO4A2 | nd | ++ | nd | ++* | ++ | (+) | − | − |
| *Rhodococcus qingshengii* AO4C3 | nd | ++* | − | − | (+) | − | ++ | + |
| *Nakamurella intestinalis* BG36 | nd | ++ | nd | ++ | nd | ++* | + | + |
| *Sphingobacterium* spp. BG19 (OTU10) | nd | ++* | nd | ++ | ++ | − | nd | ++ |
| *Sphingobacterium* spp. BG35 (OTU10) | nd | ++ | nd | ++ | nd | ++* | nd | ++ |
| *Sphingobacterium* spp. BG48 (OTU10) | nd | ++ | nd | ++ | ++ | − | nd | ++* |
| *Chryseobacterium lactis* AO5P3 | nd | ++ | nd | ++ | nd | ++* | nd | ++ |
| *Enterococcus faecalis* AO2T2 (OTU7) | nd | + | − | − | ++ | − | nd | ++* |
| *Brevundimonas vesicularis* AO5C4 (OTU5) | nd | ++* | nd | ++ | nd | ++ | − | − |
| *Ochrobactrum anthropi* AO5P2 | nd | ++ | nd | ++ | nd | ++* | − | − |
| *Pandoraea fibrosis* AO4A4 | nd | + | nd | +* | nd | + | nd | + |
| *Ottowia* spp. A1-ST13 | nd | ++* | − | − | − | − | (+) | − |
| *Acinetobacter guillouiae* AO3A2 (OTU2) | nd | ++ | nd | ++* | nd | ++ | nd | ++ |
| *Acinetobacter albensis* AO5C2 (OTU3) | nd | +* | nd | + | nd | + | nd | + |
| *Acinetobacter albensis* A5-ST23 (OTU3) | nd | +* | − | − | (+) | − | nd | + |
| *Raoultella ornithinolytica* AO4A1 (OTU15) | + | (+) | nd | +* | nd | ++ | nd | ++ |
| *Aeromonas media* AO5C3 | nd | ++* | nd | ++ | nd | ++ | − | − |
| *Stenotrophomonas lactitubi* BG28 | nd | ++* | nd | ++ | nd | ++ | nd | ++ |
